# Overviews of systematic reviews: great promise, greater challenge

**DOI:** 10.1186/s13643-017-0582-8

**Published:** 2017-09-08

**Authors:** Joanne E. McKenzie, Sue E. Brennan

**Affiliations:** 1 School of Public Health and Preventive Medicine, Monash University, The Alfred Centre, 99 Commercial Road, Melbourne, VIC 3004 Australia; 2 Cochrane Australia, School of Public Health and Preventive Medicine, Monash University, Melbourne, Australia

## Abstract

The proliferation of systematic reviews and escalating demand from policy makers has driven a newer form of evidence synthesis—overviews of systematic reviews. *Systematic Reviews* are publishing a special thematic series on overviews and are encouraging submissions on the development and evaluation of methods for this review type. The authors’ of this editorial introduce the series by considering challenges that arise when conducting an overview and what methods guidance is available. They emphasise the importance of evaluating overview methods to understand the trade-offs of using different approaches and propose that a more systematic and coordinated approach to methods development would be beneficial. Finally, they consider the potential for overviews to drive improvements in the conduct and reporting of systematic reviews.

## Introduction

With over 20,000 controlled trials of healthcare interventions published annually [1], systematic reviews are a necessary tool for making vast bodies of research accessible. Since their debut in medical journals around 30 years ago, the publication rate of systematic reviews has rapidly accelerated [2]. In 2014, it was estimated that more than 8000 systematic reviews were indexed annually on MEDLINE, a threefold increase over the last decade [3]. This growth in systematic review production, along with escalating demand from policy makers for rapid reviews of research, has driven an increase in a newer form of synthesis—overviews of systematic reviews—which is the focus of this series.

Overviews involve the systematic retrieval and identification, assessment of bias and integration of results from multiple systematic reviews [4–6]. They have the potential to confer many benefits and opportunities. Notably, overviews capitalise on previous research synthesis efforts bringing efficiencies that may lessen research waste. While in and of itself this has clear benefit, it should also enable broader evidence synthesis questions to be addressed (which may not be possible within the confines of limited resources available for systematic reviews) and in a faster timeframe. Ingredients in realising these benefits include the availability of well-conducted and reported systematic reviews (a point which we return to in the conclusion) and methods to deal with the many issues that arise in undertaking overviews.

While the distinguishing feature of overviews is that the information is compiled from systematic reviews, rather than primary studies, their purposes vary, as does the terminology used to describe them [7]. The purposes of overviews include (but are not limited to) mapping the available evidence [4], examining the effects of different interventions for the same condition or population [8], examining the effects of the same intervention for different conditions or populations (also referred to as multiple-indication reviews) [8, 9] or examining reasons for discordance of findings and conclusions across reviews [10]. Overviews are more suited to some purposes than others, and careful consideration of whether they are the appropriate type of review (overview or systematic review of primary studies) is required [11].

## What unique issues arise in overviews?

It is not uncommon for overviews to be viewed as a straightforward extension of their well-established pre-cursor, the systematic review of primary studies. Consequently, experienced review authors may anticipate that overviews will present familiar challenges to which they can apply their existing repertoire of methods. In many regards this is true; however, unique issues arise in overviews that require methodological solutions for which we have no exact parallel in a review of primary studies. Many of these issues stem from alignment (or lack thereof) between the overview question and the questions addressed by the included reviews, and the conduct and reporting of systematic reviews.

**Fig. 1:**
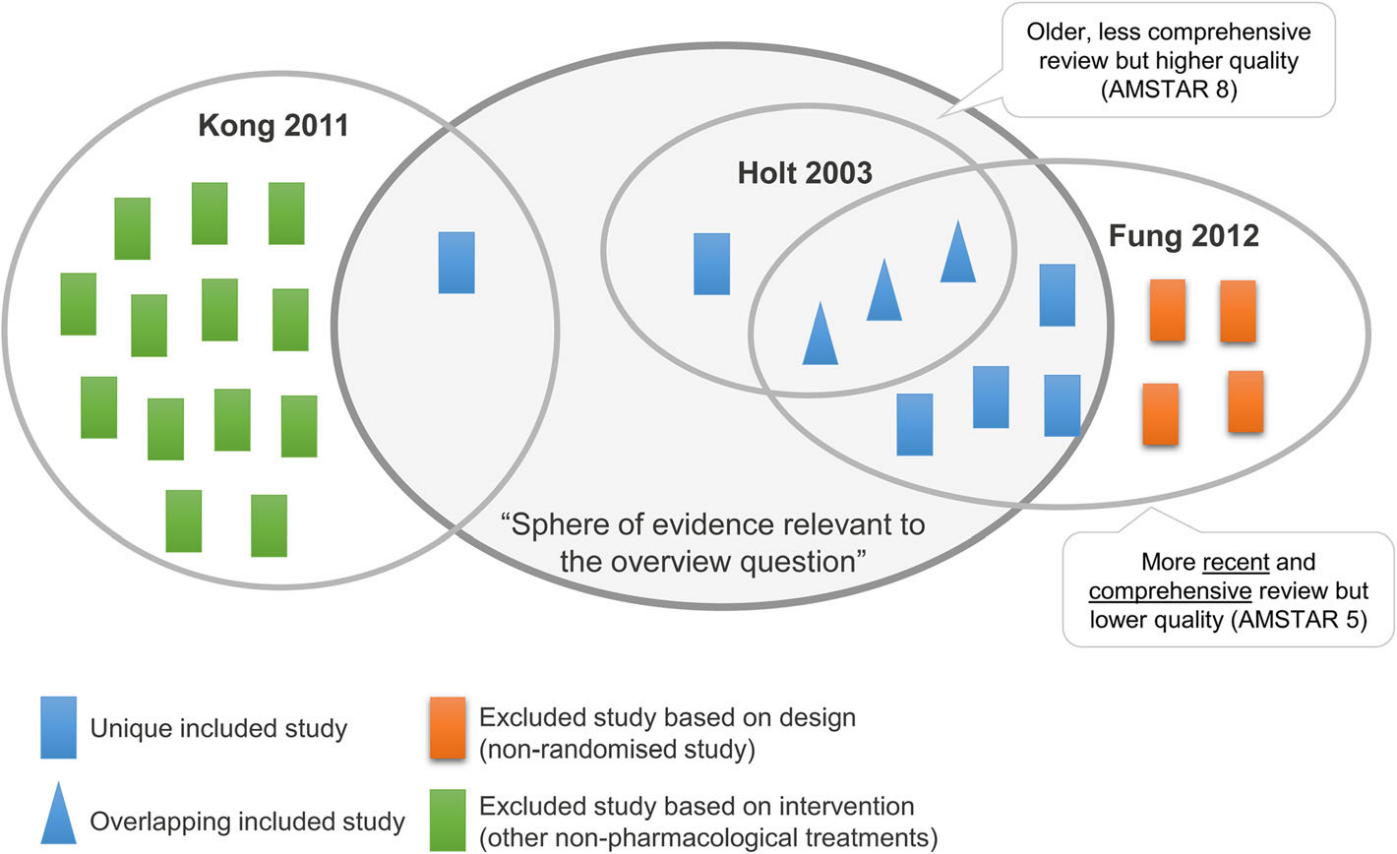
Alignment and overlap of systematic reviews and their included primary studies in an overview of aromatherapy

Chief amongst these issues is ‘overlap’. Overlap is shorthand for when the same studies (and data) appear in more than one included systematic review (Fig. 1). The simplest solution to overlap is to include only one systematic review (or meta-analysis) addressing each question. But reviews rarely address identical questions, and selecting one review from multiple can result in loss of important data or entire studies. The alternative, which is to include multiple reviews addressing the same or similar question, can have benefits but brings additional complexity. Benefits include providing a more complete picture of relevant evidence and an explicit basis from which to examine discordant results or conclusions across reviews. Complexity arises if re-analysis is required to include all relevant studies (or exclude ineligible studies) and to ensure overlapping studies do not receive too much weight. Such efforts may be stymied if data from primary studies are missing, inadequate or inconsistently reported in systematic reviews [6]. Similar issues arise with information required to interpret studies, for example, when primary study characteristics and risk of bias assessments are incompletely reported [4]. Overlaying these issues is the risk of bias introduced through the conduct and reporting of systematic reviews (including when reviews are not updated) [12, 13]. Each of these issues requires methodological solutions, for which overview authors need to plan.

## What is the status of methods guidance for overviews?

Ten years on from publication of the first methods guidance for conducting overviews [8], recent systematic reviews provide a timely synthesis of current guidance [6, 14, 15]. These reviews found inconsistent guidance and a lack of operational detail required to apply methods. Multiple methods options were identified for dealing with issues such as overlap, incomplete data, and reviews with questionable methods or problematic reporting. There is a sense, however, that many methods are less the product of coordinated development than they are a reflection of the emergent ‘methodological template[s]’ ([10] p. 446) used by author teams to tackle the issues that confronted them. There are exceptions, such as the coordinated development of tools to appraise systematic reviews, namely AMSTAR and ROBIS [13, 16, 17]. But even here, the science stops short of providing guidance on how to integrate these assessments when interpreting findings using methods such as GRADE (a gap noted in all reviews of guidance). The reviews of guidance, and other methods studies in this series, bring into focus the need for a coordinated approach to methods development, sensitive to the different purposes of overviews and contexts in which they are performed, and the need for evaluation to understand the trade-offs of choosing one method over another.

## What evidence do we need to understand the performance of these methods?

The choice of methods used in overviews may affect the trustworthiness of the findings, coverage of the evidence, and usability and usefulness of the overview, amongst other outcomes. Decisions as to which methods to use are best informed by methods research [18], along with theoretical considerations. For example, research comparing different search filters to identify systematic reviews allows us to determine which is preferable based on metrics such as sensitivity and precision (e.g. [19]), whereas audits of overviews allow us to identify methods being used in practice, and where improvements in conduct and reporting may be required (e.g. [5, 20, 21]). Other potentially valuable research includes examining the impact of different methodological eligibility criteria (e.g. include all systematic reviews, include only systematic reviews at low risk of bias) on outcomes such as the overview’s findings and coverage of available studies. Another example involves examining the impact of retrieving primary studies to extract information missing from the systematic review (e.g. risk of bias assessment for a study), or where discrepant information about a study is reported across systematic reviews. Having a comprehensive understanding of the available methods, what evaluations are available and where there are gaps, may help inform and prioritise where methods evaluations are necessary [22].

## Conclusions

While development and evaluation of methods for overviews is necessary, this effort needs to happen in tandem with improvements in the conduct and reporting of systematic reviews. Herein lies the opportunity for overviews to drive these improvements, analogous to the way in which systematic reviews have driven improvements in the conduct and reporting of primary studies. Ultimately, producing more reliable, valid, and complete overviews requires comprehensive coverage of evidence within an area and greater standardisation of systematic review methods. To achieve this, we need coordination amongst review teams examining different parts of a broad evidence synthesis question. Registration of systematic reviews through PROSPERO—an international prospective register of systematic reviews—could play an important role in this coordinated effort through the linking of review teams.
